# Supplementary material for: MeCP2 regulates Tet1-catalyzed demethylation, CTCF binding, and learning-dependent alternative splicing of the BDNF gene in Turtle
Source: eLife. 2017 Jun 8;6:e25384. doi: 10.7554/eLife.25384 (PMC5481183; doi:10.7554/eLife.25384)
Supplement: Supplementary file 1. — Sequence data from PCR products generated from analysis of the splice site shown in Figure 1C. Sequence from the single band produced from genomic DNA is shown in sample 1. Data from cDNA produced two PCR bands whose sequences are shown for the upper band (sample 2) and the lower band (sample 3). ^Indicates the splice site in sample 3. DOI: http://dx.doi.org/10.7554/eLife.25384.010 [file elife-25384-supp1.docx]

**Figure 1 – figure supplement 1. Raw sequence data from splice site PCR and RT-PCR.**

Genomic DNA Sample 1:

GGTTACACAAAAGAGGGTTGCAGGGGCATAGACAAGAGGCACTGGAATTC

CCAGTGCCGAACTACCCAGTCTTATGTGCGAGCTCTCACCATGGATAACA

AAAAGAGAGTTGGCTGGCGGTTTATAAGAATAGACACTTCCTGTGTATGT

ACATTGACCATTAAAAGGGGAAGA

cDNA Upper Band Sample 2:

GGTTACACAAAAGAGGGTTGCAGGGGCATAGACAAGAGGCACTGGAATTC

CCAGTGCCGAACTACCCAGTCTTATGTGCGAGCTCTCACCATGGATAACA

AAAAGAGAGTTGGCTGGCGGTTTATAAGAATAGACACTTCCTGTGTATGT

ACATTGACCATTAAAAGGGGAAGA

cDNA Lower Band Sample 3:

GGTTACACAAAAGAGGGTTGC^CTACCCAGTCTTATGTGCGAGCTC

TCACCATGGATAACAAAAAGAGAGTTGGCTGGCGGTTTATAAGAATAGAC

ACTTCCTGTGTATGTACATTGACCATTAAAAGGGGAAGA
